# Improved visualization of hepatic tumors in magnetic resonance–guided thermoablation using T1-inversion-recovery imaging with variable inversion time

**DOI:** 10.1007/s00330-023-09696-9

**Published:** 2023-05-03

**Authors:** J. Kübler, P. Krumm, P. Martirosian, M. T. Winkelmann, G. Gohla, K. Nikolaou, R. Hoffmann

**Affiliations:** 1 Department of Diagnostic and Interventional Radiology, University Hospital of Tübingen, Tübingen, Germany; 2 Section On Experimental Radiology, University Hospital of Tübingen, Tübingen, Germany

**Keywords:** Interventional radiology, Ablation techniques, Magnetic resonance imaging, Liver, Image enhancement

## Abstract

**Objectives:**

In magnetic resonance (MR)–guided interventions, visualization of hepatic lesions may be difficult using standard unenhanced T1-weighted gradient-echo volume-interpolated breath-hold (VIBE) sequence due to low contrast. Inversion recovery (IR) imaging may have the potential to improve visualization without the necessity to apply contrast agent.

**Methods:**

Forty-four patients (mean age 64 years, female 33%) scheduled for MR-guided thermoablation due to liver malignancies (hepatocellular carcinoma or metastases) were prospectively included in this study between March 2020 and April 2022. Fifty-one liver lesions were intra-procedurally characterized before treatment. Unenhanced T1-VIBE was acquired as part of the standard imaging protocol. Additionally, T1-modified look-locker images were acquired with eight different inversion times (TI) between 148 and 1743 ms. Lesion-to-liver contrast (LLC) was compared between T1-VIBE and IR images for each TI. T1 relaxation times for liver lesions and liver parenchyma were calculated.

**Results:**

Mean LLC in T1-VIBE sequence was 0.3 ± 0.1. In IR images, LLC was highest at TI 228 ms (1.04 ± 1.1) and significantly higher compared to T1-VIBE (*p* < 0.001). In subgroup analysis, lesions of colorectal carcinoma showed the highest LLC at 228 ms (1.14 ± 1.4), and hepatocellular carcinoma showed the highest LLC at 548 ms (1.06 ± 1.16). T1-relaxation times in liver lesions were higher compared to the adjacent liver parenchyma (1184 ± 456 vs. 654 ± 96 ms, *p* < 0.001).

**Conclusions:**

IR imaging is promising to provide improved visualization during unenhanced MR-guided liver interventions compared to standard T1-VIBE sequence when using specific TI. Low TI between 150 and 230 ms yields the highest contrast between liver parenchyma and malignant liver lesions.

**Clinical relevance statement:**

Improved visualization of hepatic lesions during MR-guided percutaneous interventions using inversion recovery imaging without the necessity to apply contrast agent.

**Key points:**

*• Inversion recovery imaging is promising to provide improved visualization of liver lesions in unenhanced MRI.*

*• Planning and guidance during MR-guided interventions in the liver can be performed with greater confidence without necessity to apply contrast agent.*

*• Low TI between 150 and 230 ms yields the highest contrast between liver parenchyma and malignant liver lesions.*

**Supplementary Information:**

The online version contains supplementary material available at 10.1007/s00330-023-09696-9.

## Introduction

Percutaneous thermoablations have become a widely accepted treatment option for malignancies of the liver [1, 2]. Whereas ultrasound and computed tomography are more commonly available modalities for image guidance, magnetic resonance (MR)–guided imaging offers several advantages such as real-time fluoroscopy, freely selectable image planes, high soft-tissue contrast, and immediate confirmation of success through illustration of the ablation zone at the end of the procedure [3]. On these grounds, percutaneous thermoablations of hepatic lesions are almost exclusively performed under MR guidance at our institution, if the patient allows [4–6]. Standard protocol for diagnostic imaging of the liver prior to interventions usually contains dynamic contrast-enhanced sequences after application of gadolinium-based contrast agents (GBCA) [7–10]. However, in MR-guided interventions, contrast-enhanced imaging is preferably performed after completion of the ablation for visualization of the ablation zone, and differentiation between necrotic tissue and vital parenchyma as well as detection of vessel injuries and bleeding [11–13]. Therefore, unenhanced imaging sequences are commonly used for treatment planning and guidance of the applicator during the procedure [13, 14]. Whereas large tumors can usually be detected well even without use of GBCA, small lesions are sometimes difficult to visualize [15]. This complicates intraparenchymal navigation of the applicator and bears the risk of insufficient ablation of the liver lesion. Therefore, it is beneficial to use MR sequences that provide high contrast between the targeted liver lesion and the surrounding liver parenchyma. Inversion recovery (IR) imaging exploits the feature of different T1-relaxation times in different tissues and is commonly used in clinical routine for suppression of fat (short tau inversion recovery (STIR)) or fluid (fluid attenuated inversion recovery (FLAIR)) [16]. Since inversion time (TI) can be adapted individually, IR imaging also provides the opportunity to suppress signal other than fat or water, causing a high contrast between tissues as applied in cardiac MR imaging for scar visualization [17, 18].

In this study, we investigated the potential of T1-inversion recovery imaging with different TI for improved visualization of hepatic lesions in unenhanced MRI compared to standard unenhanced T1 volume interpolated breath-hold examination (VIBE).

## Materials and methods

### Study population

This prospective study was approved by the Institutional Review Board of the University of Tübingen. Between January 2020 and April 2022, patients scheduled for MR-guided microwave ablation for treatment of hepatic malignancies were prospectively included. Locally ablative treatment was indicated by the interdisciplinary tumor conference of the clinic. All patients had received contrast-enhanced liver MR imaging according to the local standard protocol prior to thermoablation within 4 to 6 weeks, for image-based diagnosis of a malignant hepatic lesion. Malignancy of the hepatic lesion was either pathologically confirmed or clinically highly probable due to patient history and a primary tumor known for hepatic metastatic spread.

### Interventional device

Subsequent locally ablative treatment of liver lesions in the context of this study was performed with a MR-compatible therapeutic microwave antenna (Disposable Microwave Therapeutic Antenna, 14G, 15 cm, Nanjing ECO Microwave System Co.).

### Imaging protocol

MR imaging was performed on a 1.5-T scanner (Aera, Siemens Healthineers) equipped with a 32-channel body-phased-array coil.

Patients underwent standard imaging protocol for hepatic intervention. This protocol included a T2-weighted half Fourier single-shot turbo spin-echo sequence (HASTE, TR = 1100 ms, TE = 94 ms, FA = 160°, BW = 488 Hz/pixel, ST = 3 mm, FOV = 380 × 380 mm, matrix = 320 × 320), a fat-saturated (FS) T2-weighted STIR sequence (TR = 1400 ms, TE = 81 ms, FA = 160°, TI = 180 ms, BW = 449 Hz/pixel, ST = 6 mm, FOV = 340 × 340 mm, matrix = 384 × 384), and a fat-saturated unenhanced 3D T1-weighted VIBE sequence (TR = 3.5 ms, TE = 1.4 ms, FA = 10°, BW = 400 Hz/pixel, ST = 2 mm, FOV = 340 × 340 mm, matrix = 256 × 256), which was repetitively performed during intervention to confirm correct position of the applicator after repositioning. For tumor targeting, a MR-fluoroscopic sequence in three imaging orientations was used (BEAT-Multislice multiplanar interactive real-time sequence, TR = 464 ms, TE = 3.2 ms, FA = 20°, BW = 500 Hz/pixel, ST = 8 mm, matrix = 128 × 128).

Additionally, patients received a single-slice Modified Look-Locker Inversion Recovery (MOLLI) T1-mapping sequence prior to insertion of the applicator covering the largest tumor diameter. The product type MOLLI sequence (MyoMaps, vendor label T1long, 5(3)3 pulse scheme, Siemens Healthineers) was applied without ECG gating with several TIs between 148 and 1743 ms. Other sequence parameters were as follows: TR = 398.4 ms, TE = 1.08 ms, FA = 35°, TI = 148 ms, 228 ms, 548 ms, 628 ms, 946 ms, 1025 ms, 1343 ms and 1743 ms, BW = 1085 Hz/pixel, ST = 8 mm, FOV = 400 × 400 mm, matrix = 256 × 256.

After completion of the ablation and extraction of the applicator, GBCA-enhanced fat-saturated T1-VIBE was acquired to demarcate the ablation zone, confirm successful treatment, and exclude complications such as bleeding.

### Image analysis

Images were analyzed on standard workstations (Centricity PACS, RA1000, General Electrics). Liver lesions were identified in unenhanced sequences as T1-hypointense relative to the surrounding liver parenchyma and moderately hyperintense on T2-fat saturated sequence. Additionally, images were compared with previously acquired contrast-enhanced MRI.

Signal intensity (SI) of the liver (SI liver) and liver lesions (SI lesion) were measured for quantitative evaluation of contrast. A circular region of interest (ROI) was manually placed within the lesion and the adjacent liver parenchyma. The ROI was drawn as large as possible avoiding vessels, bile ducts, necrotic portions, and artifacts, if present. Care was taken to achieve consistent positioning of a ROI across different series within the same patient. Lesion-to-liver contrast (LLC) was calculated according to the following formula:

$$\mathrm{LLC}=\left.\left| \frac{\mathrm{SI Liver }-\mathrm{ SI Lesion}}{\mathrm{SI Liver}}\right.\right|$$

### Statistical analysis

Data were analyzed using JMP (Version 14.2.0, SAS Institute Inc.) and SPSS 22.0 (IBM Corp.). Continuous variables are expressed as mean value ± standard deviation (SD) if not declared otherwise. Normal distribution of parameters was assessed visually in curves using Saphiro–Wilk test [19]. Two-sided *t*-tests on paired differences were applied for normally distributed variables. For non-normally distributed independent variables, a Kruskal–Wallis test; for dependent samples, a Wilcoxon rank sum test; or in case of more than two levels of repeated measurements, a Friedman test was used. Post hoc Dunn-Bonferroni tests ensued with proper alpha correction. Statistical significance was set for *p* ≤ 0.05.

## Results

Between January 2020 and April 2022, 44 patients with 51 hepatic lesions were included. Mean age of patients was 64 ± 13 years. Most patients were male (29 patients, 66%). Most hepatic lesions were metastases of colorectal carcinoma (20 lesions, 39%), followed by hepatocellular carcinoma (12 lesions, 24%), metastases of malignant melanoma (9 lesions, 18%), breast cancer (5 lesions, 10%), neuroendocrine tumor (2 lesions, 4%), pancreatic tumor (1 lesion, 2%), cholangiocellular carcinoma (1 lesion, 2%), and uveal melanoma (1 lesion, 2%). Most lesions were located in hepatic segment VIII (16 lesions, 32%), followed by segment VII (11 lesions, 22%), segment IV (7 lesions, 14%), segment V (6 lesions, 12%), segment VI (5 lesions, 10%), segment II (4 lesions, 8%), and segment III (6 lesions, 12%). Mean size of liver lesions was 19.1 mm with a total range between 6 and 41 mm. During contrast-enhanced imaging at the end of the procedure, all lesions were entirely covered by the ablation zone without evidence of residual tumor, corresponding to a technical success rate of 100%. Characteristics of patients and lesions are summarized in Table 1.

**Table 1:** Characteristics of patients and lesions. *CRC* colorectal carcinoma, *HCC* hepatocellular carcinoma

	Patients (*n* = 44)	Lesions (*n* = 51)
No. of patients with > 1 lesion treated	6	–
Age—mean (total range)—[year]	63.8 (28–86)	–
Male sex—frequency	29 (66%)	–
Primary cancer—no		
CRC	15 (34%)	20 (39%)
HCC	12 (27%)	12 (24%)
Malignant melanoma	9 (20%)	9 (18%)
Breast cancer	3 (7%)	5 (10%)
Neuroendocrine tumor	2 (5%)	2 (4%)
Pancreatic tumor	1 (2%)	1 (2%)
Cholangiocellular carcinoma	1 (2%)	1 (2%)
Uveal melanoma	1 (2%)	1 (2%)
Liver segment location—frequency		
II	–	4 (8%)
III	–	2 (4%)
IV a/b	–	7 (14%)
V	–	6 (12%)
VI	–	5 (10%)
VII	–	11 (22%)
VIII	–	16 (32%)
Lesion size—mean (total range)—[mm]	–	19.1 (6–41)

### Image analysis of liver lesions

**Fig. 1:**
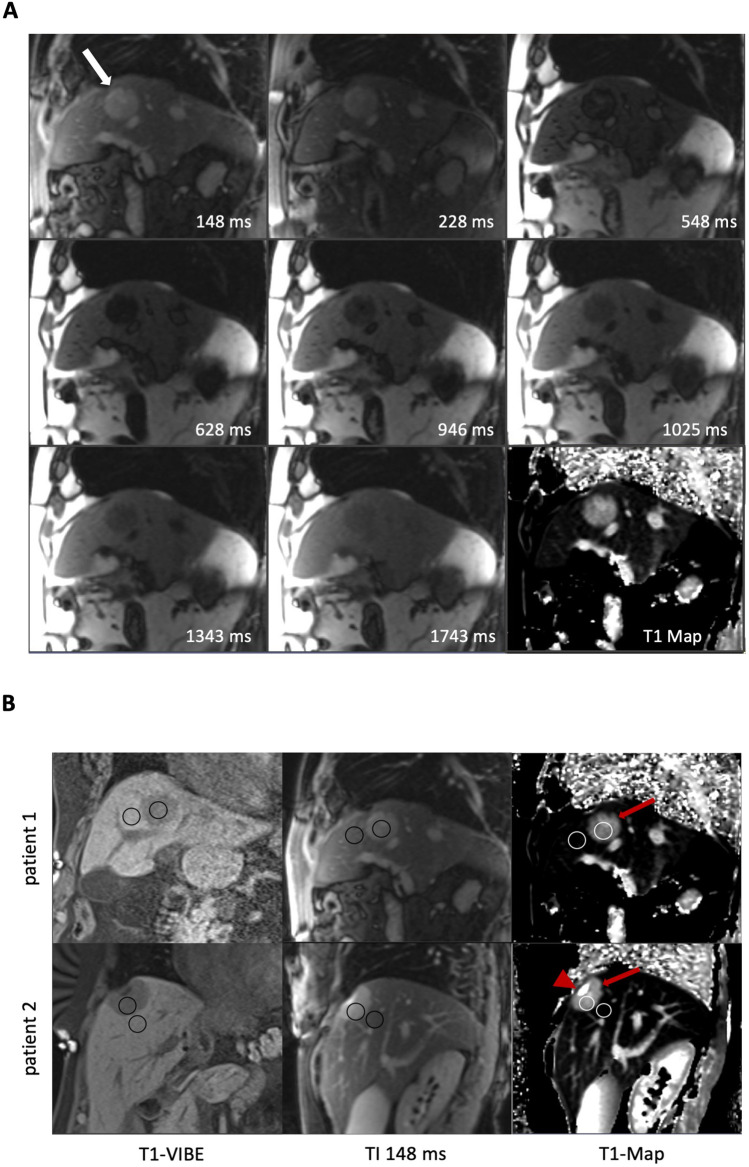
**A** Look-Locker IR sequences in sagittal orientation with TI between 148 and 1743 ms and T1-Map. The arrow in the first image points at a metastasis of colorectal carcinoma that was treated in the subsequent thermoablation. Signal intensities of the liver lesions and the adjacent liver parenchyma vary depending on TI. **B** Examples of ROI placement in two different patients for analysis of signal intensity and T1 relaxation times. The first ROI was placed in the center of the liver lesion (arrow in T1-Map) carefully avoiding necrotic portions, if present (arrowhead in T1-Map). A second ROI of similar size was placed in the adjacent liver parenchyma avoiding vessels or bile ducts

**Fig. 2:**
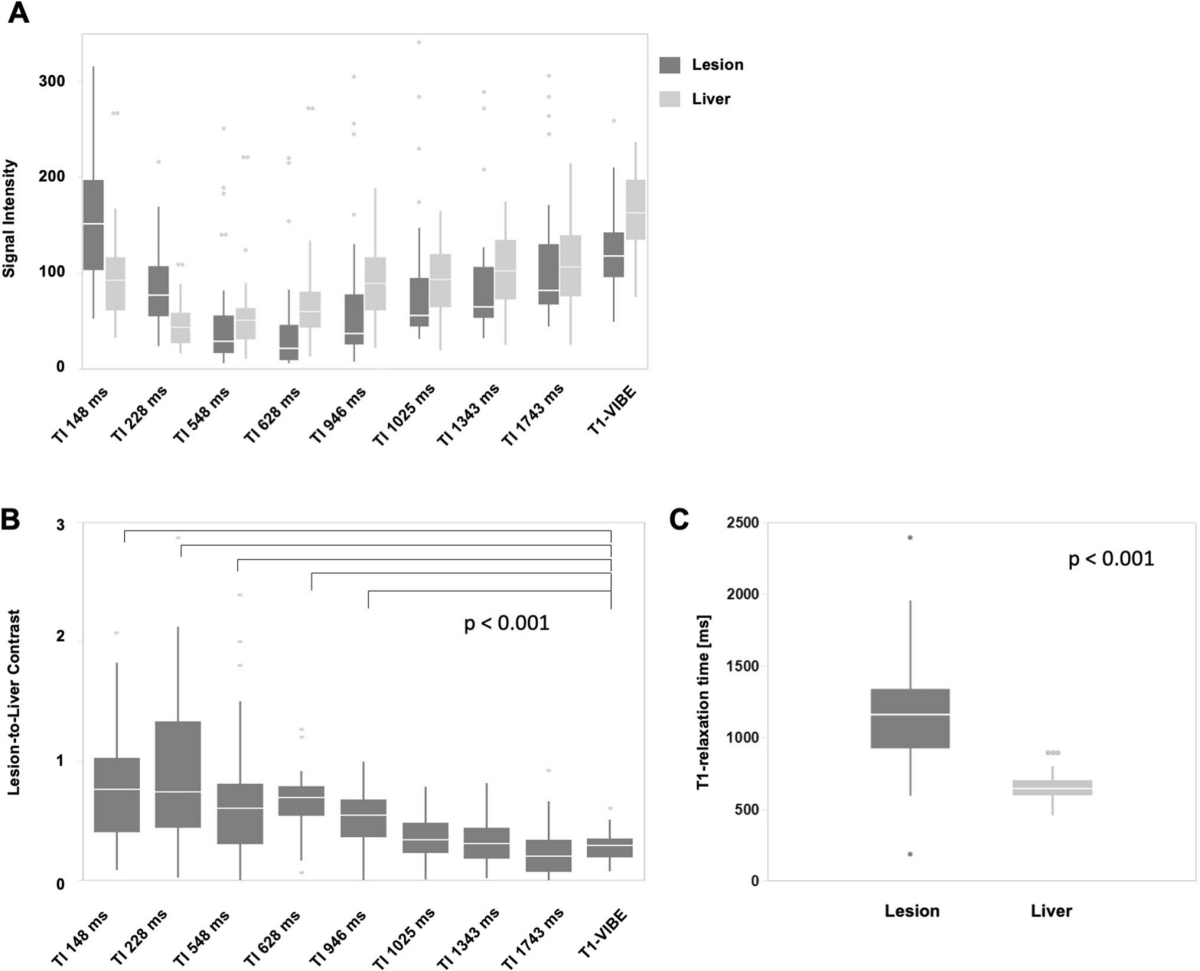
Signal intensity, lesion-to-liver contrast, and T1 values. **A** Signal intensity of liver lesions and adjacent liver parenchyma was measured in Look-Locker IR images with TI between 148 and 1743 ms and in standard T1-VIBE sequence. Calculated LLC is shown in **B** for different TI and T1-VIBE, respectively. **C** Absolute T1-relaxation time was significantly higher in liver lesions compared to adjacent liver parenchyma

LLC of 51 liver lesions was analyzed in standard T1-VIBE images and Look-Locker IR images with TI between 148 and 1743 ms (Fig. 1 and Fig. 2A). Including all lesions, calculated LLC was highest in IR images with a TI of 228 ms (mean LLC 1.04 ± 1.1) and lowest at high TI of 1743 ms (mean LLC 0.24 ± 0.2). Mean LLC in standard T1-VIBE sequence was 0.29 ± 0.11 and lower compared to IR images with TI between 148 and 1343 ms. The difference in LLC was statistically significant in TI 148 ms (mean LLC 0.87 ± 0.75, *p* < 0.001), TI 228 ms (mean LLC 1.0 ± 1.1, *p* < 0.001), TI 548 ms (mean LLC 0.71 ± 0.72,* p* < 0.001), TI 628 ms (mean LLC 0.65 ± 0.23, *p* < 0.001), and TI 946 ms (mean LLC 0.51 ± 0.24, *p* < 0.001). Differences in LLC are depicted in Fig. 2 B.

T1 relaxation times were higher in liver lesions (mean T1: 1187 ± 456 ms) compared to the adjacent liver parenchyma (mean T1: 654 ± 96 ms, *p* < 0.001, Fig. 2C).

**Fig. 3:**
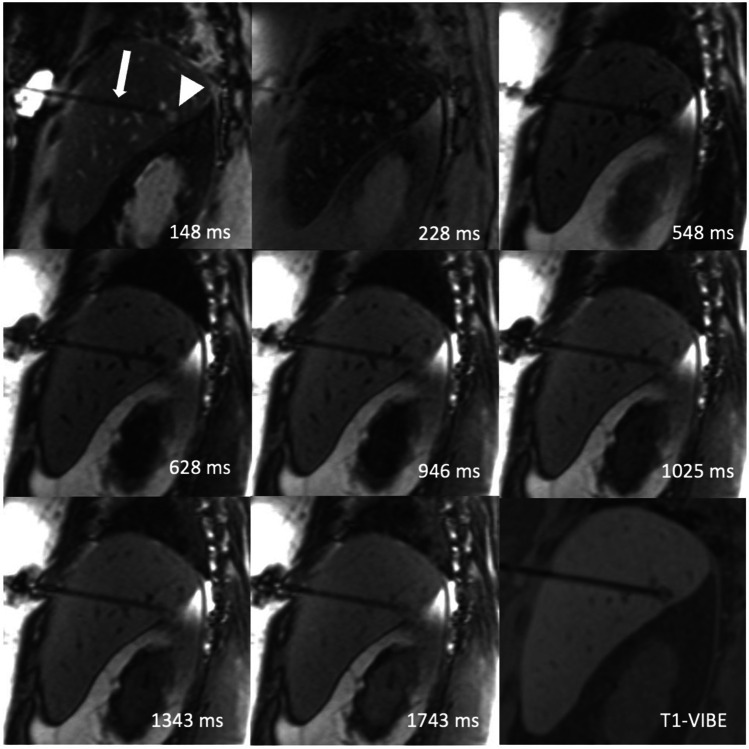
Applicator positioned in the target lesion. Look-Locker IR sequences in sagittal orientation with TI between 148 and 1743 ms and standard T1-VIBE demonstrating the positioning of a microwave ablation antenna (arrow in the first image) in a liver metastasis (arrowhead in the first image). Images were acquired before initiation of treatment. The applicator is visible with homogenously low signal intensity and does not obscure the target lesion

The applicator caused a susceptibility artifact with consistent appearance at each TI that does not interfere with navigation or obscure the target lesion (Fig. 3).

### Image analysis of subgroups

**Fig. 4:**
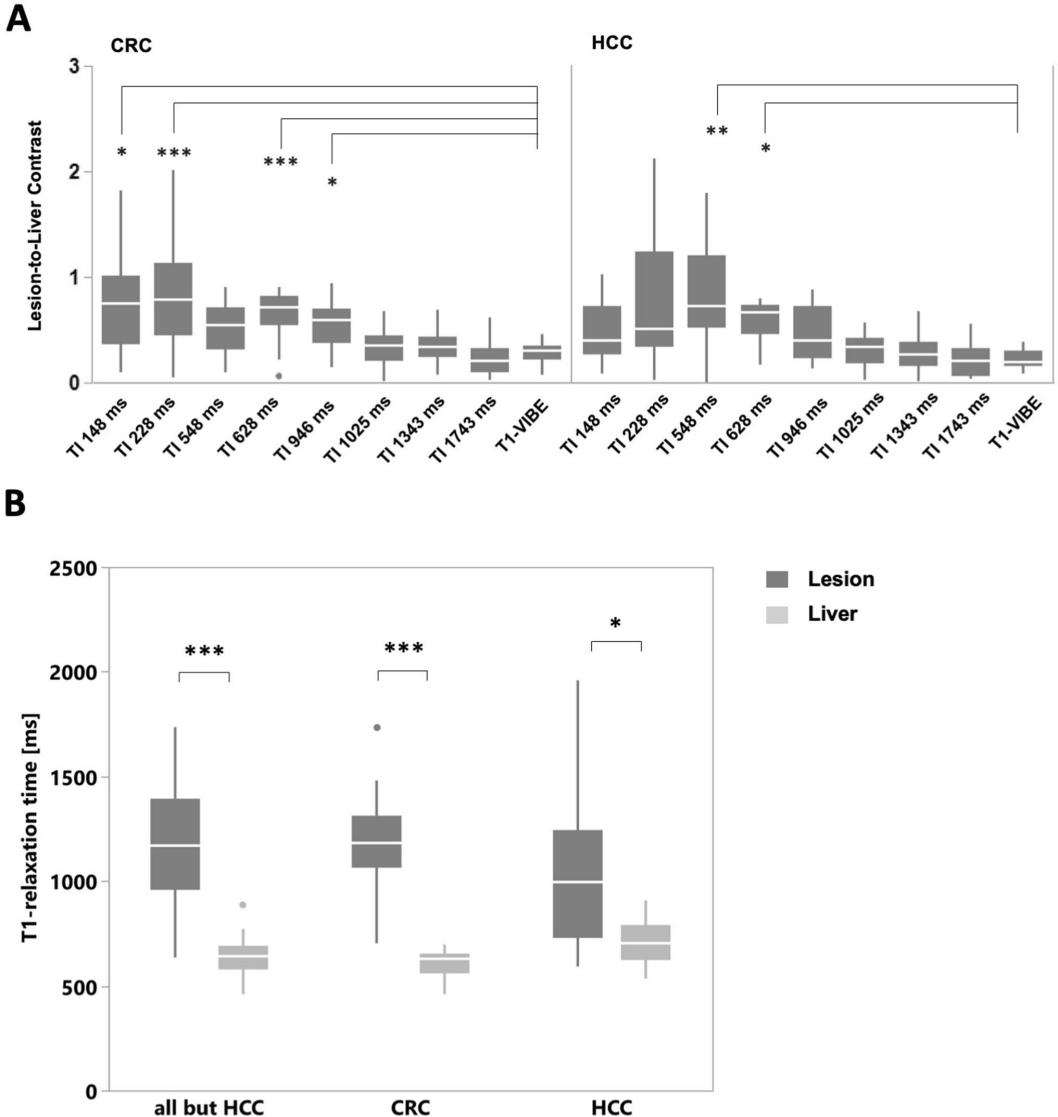
LLC and T1 values in subgroups. **A** LLC in CRC and HCC lesions for different TI and T1-VIBE sequence, respectively. **B** T1 values in lesions and adjacent liver parenchyma. HCC, hepatocellular carcinoma; CRC, colorectal carcinoma; LLC, lesion-to-liver contrast

Subgroups with lesions of HCC and CRC were further analyzed (Fig. 4).

In HCC lesions, the highest LLC was seen in IR images with TI 548 ms (mean LLC 1.06 ± 1.16), and lowest was seen in 1743 ms (mean LLC 0.21 ± 0.16), which was close to LLC in standard T1-VIBE (0.22 ± 0.09). Compared to T1-VIBE, significantly higher LLC was measured in Look-Locker images with TI 548 ms (*p* = 0.007) and 628 ms (mean LLC 0.59. ± 0.20, *p* = 0.029).

In CRC lesions, the highest LLC was measured in IR images with TI 228 ms (mean LLC 1.14. ± 1.4) and lowest in 1743 ms (mean LLC 0.27 ± 0.22). Mean LLC in T1-VIBE was 0.29 ± 0.1. Compared to T1-VIBE, significantly higher LLC was measured in 148 ms (mean LLC 0.98. ± 0.89, *p* = 0.032), 228 ms (*p* < 0.001), 628 ms (mean LLC 0.64. ± 0.23, *p* < 0.001), and 946 ms (mean LLC 0.50. ± 0.25, *p* = 0.049).

A small number of the liver lesions (*n* = 9) investigated in our study were caused by malignant melanoma with partially hyperintense lesions in T1-VIBE sequence. However, LLC of melanoma lesions was similar compared to other lesions without statistically significant differences (Supplemental 1).

T1 values of lesions were significantly higher compared to adjacent liver parenchyma in both subgroups as well as in the entire collective excluding HCC patients. Mean T1 relaxation time in HCC lesions was 1063 ± 381 ms compared to 712 ± 115 ms (*p* = 0.02), in CRC lesions 1319 ± 545 ms compared to 609 ± 69 ms (*p* < 0.001), and in all lesions excluding HCC patients 1225 ± 474 ms compared to 636 ± 84 ms (*p* < 0.001). Patients with HCC lesions showed higher T1-relaxation times of the liver parenchyma (T1 liver_HCC_ = 712 ± 115 ms) compared to the subgroup with CRC lesions (T1 liver_CRC_ = 609 ± 69 ms, *p* = 0.035) and the entire collective excluding HCC patients (T1 liver_all but HCC_ = 636 ± 84 ms, *p* = 0.15).

## Discussion

This study systematically compared lesion to liver contrast in unenhanced T1-VIBE and T1-modified Look-Locker IR images for better unenhanced intraprocedural lesion delineation in MR-guided thermoablation. With low TI (150–230 ms) Look Locker IR images, significantly higher contrast between liver parenchyma and malignant liver lesions was achieved.

### Evaluation of contrast

For objective evaluation of contrast, we calculated LLC in a sequence of magnitude reconstructed Look-Locker images with TI between 148 and 1743 ms and compared them to standard T1-VIBE sequence. Contrast between liver lesions and liver parenchyma was depending on TI and significantly higher in IR images with TI between 148 and 946 ms, but also associated with higher variance in contrast. At relatively low TI of 148 and 228 ms, the highest contrast was achieved. In these images, signal intensity of the liver parenchyma was lower compared to the signal intensity of liver lesions. Suppression of the signal of the liver parenchyma was achieved with TI between 228 and 548 ms. Signal of hepatic lesions was lowest with TI between 548 and 628 ms. With TI longer than 948 ms, contrast decreased again and was similar or even lower compared to T1-VIBE. Fahlenkamp et al. investigated a similar approach for improved detection of small hepatic lesions and suggested a TI of 480 ms for signal suppression of hepatic metastases. However, they investigated a different setup with phase-sensitive reconstructed IR images in gadoxetate disodium-enhanced scans [20].

When aiming at full signal suppression with IR imaging similar to fat suppression in STIR or fluid suppression in FLAIR, two approaches are possible in the liver. Either signal suppression of hepatic lesions or signal suppression of liver parenchyma. T1 relaxation times of liver lesions are higher compared to the adjacent liver parenchyma due to inherent structural differences of different tissues.

In subgroup analysis of the two most frequent tumor entities of our patient collective, we noticed slightly elevated T1 values in liver parenchyma of HCC patients compared to parenchyma of non-HCC patients. This might be ascribed to liver cirrhosis which is known to increase T1 values [21, 22]. Overall differences in T1 values of liver parenchyma between different subgroups were minor compared to high T1 values of malignant lesions. Therefore, adjustment of TI dependent on structural changes of the liver parenchyma for maximizing contrast might be negligible. Results of our subgroup analysis were similar compared to the analysis of the entire collective. T1-VIBE resulted in consistently low LLC, whereas IR images provided higher contrast with TI shorter than 1000 ms. However, differences in statistical significance should be assessed with care due to the small sample size of our subgroups.

### Visibility of the interventional device

To ensure safe navigation during MR-guided interventions, the applied device needs to be visible. In this study, we prospectively included patients that were scheduled for thermoablation of malignant liver lesions. Therefore, a MR-compatible microwave ablation system, which is routinely used at our institution, was utilized in the context of this study. The device is made of a titanium-alloy shaft and an active ceramic tip. With each TI of the Look-Locker sequence, the applicator could be visualized and the susceptibility artifacts did not obscure the target lesions or interfere with safe navigation. However, since susceptibility artifacts in MR images are strongly dependent on the design of the device, there might be differences between several products.

### MR-guidance sequences and potential of inversion recovery imaging

Over the past two decades, technical improvements provided powerful tools for MR-guided interventions including sequences with higher temporal and spatial resolution as well as high signal-to-noise ratio. However, there is no single MR sequence that meets all the needs at once for interventionalists. Usually, there are several phases during minimally invasive interventions with different requirements each [23]. Initially, after positioning of the patient and prior to insertion of the interventional device, unenhanced T1- and T2-weighted sequences for anatomical overview are applied similar to diagnostic imaging. Among them, fat-saturated T1-VIBE is a 3D sequence with high spatial resolution that can be repeatedly performed after repositioning of the applicator [4, 5, 24]. Another phase includes real-time tracking of the device requiring MR-fluoroscopic sequences with high temporal resolution, typically balanced steady-state free precession acquisition (BEAT-multislice). Dynamic contrast-enhanced imaging is usually applied after completion of the ablation to ensure technical success. However, inversion recovery imaging is currently not of relevance in MR-guided interventions to the best of our knowledge.

We applied Look-Locker sequences that are typically used in cardiac imaging for myocardial tissue characterization by evaluation of T1-relaxation times [25]. These sequences were applied as a part of the study protocol only before initiation of treatment. The method is based on a non-selective 180° inversion pulse and subsequent signal acquisition at different TI for calculation of T1-maps [26]. The ECG trigger inherent in the initial modified Look-Locker sequences was removed due to non-cardiac application. In our study, Look-Locker images were used as a scout to evaluate a broad range of TI for optimal contrast between liver lesions and the adjacent liver parenchyma. The single slice of a Look-Locker sequence that was performed in our study was sufficient for evaluation of signal intensity, but to be useful as a planning or targeting sequence in interventions, it will be necessary to transfer our results of contrast optimized TI into a 3D sequence with a spatial resolution comparable to a T1-VIBE.

IR sequences generally have the disadvantage of a higher specific absorption rate and lengthened scan time due to repetitive 180° pulses. However, in recent years, there have been several breakthroughs in the field of artificial intelligence leading to substantial acceleration of MR sequences [27]. Since the short TI of 150–230 ms resulted in best contrast, the additional time in a 3D IR sequence may still be acceptable. In the future, this development might also contribute to introduction of new sequences for diagnostic imaging and to for minimal invasive MR-guided interventions.

## Limitations

Our study has several limitations. The utilization of Look-Locker sequences allows for evaluation of LLC at different TI. However, we performed only one slice covering the previously known hepatic lesion sufficient for analysis of signal intensity.

We were not able to perform procedures at scanners of other vendors or other field strength. We assume that the recommended TI values should be largely independent of manufacturers, assuming identical sequence implementation. In contrast, an optimal TI will increase with increasing field strength because the T1 time of the tissue increases. For example, about 30% longer TI at a field strength of 3 Tesla is to be expected. Our results should be regarded as preparatory work for the subsequent development of an IR sequence with both sufficient spatial resolution and an acceptable acquisition time. For clinical application in MR-guided interventions, further studies are necessary that investigate ideally 3D-IR sequences with the suggested TI that ensure sufficient spatial resolution.

Furthermore, to be suitable for practical application, a potential IR sequence also needs to visualize the interventional device. In the context of this study, we used a MR-compatible microwave ablation antenna which yielded a homogenous artifact that allowed safe navigation. However, this study focused on the visualization of hepatic lesions using IR imaging and further studies might be required to evaluate the artifact of different devices.

The small sample size of our subgroups does not allow final conclusions on an optimal TI for different tumor entities. However, our results show similar TI for improvement of contrast across the entire collective regardless of the tumor entity.

Only a minority of treated liver lesions was histologically confirmed. The majority was classified as malignant based on imaging criteria and patient history. Therefore, there is a small risk of misclassification.

## Conclusions

IR imaging with different TI is promising to provide improved visualization of target lesions during MR-guided interventions compared to standard native T1-VIBE sequence. Low TI between 150 and 230 ms yields the highest contrast between liver parenchyma and malignant liver lesions.

### Supplementary Information

Below is the link to the electronic supplementary material.

Supplementary file1 (PDF 59 KB)

## Abbreviations

BW: Bandwidth
CRC: Colorectal carcinoma
ECG: Electrocardiogram
FA: Flip angle
FLAIR: Fluid attenuated inversion recovery
FOV: Field of view
FS: Fat saturated
GBCA: Gadolinium-based contrast agents
HCC: Hepatocellular carcinoma
IR: Inversion recovery
LLC: Lesion-to-liver contrast
MOLLI: Modified Look-Locker inversion recovery
MR: Magnetic resonance
ROI: Region of interest
SD: Standard deviation
SI: Signal intensity
ST: Slice thickness
STIR: Short tau inversion recovery
TE: Echo time
TI: Inversion time
TR: Repetition time
VIBE: Volume-interpolated breath-hold

## Declarations

### Guarantor

The scientific guarantor of this publication is Rüdiger Hoffmann.

### Statistics and biometry

No complex statistical methods were necessary for this paper.

### Study subjects or cohorts overlap

No study subjects or cohorts have been previously reported.

### Methodology

• prospective

• observational

• performed at one institution
