# Supplementary material for: Improved visualization of hepatic tumors in magnetic resonance–guided thermoablation using T1-inversion-recovery imaging with variable inversion time
Source: Eur Radiol. 2023 May 3;33(10):7015–24. doi: 10.1007/s00330-023-09696-9 (PMC10511564; doi:10.1007/s00330-023-09696-9)

Appendix 1: A small number of the liver lesions (n=9) investigated in our study were caused by malignant melanoma. Some lesions were hyperintense to the surrounding liver parenchyma in T1-VIBE sequence compared to all other tumor entities with a maximum SI of 259 vs. 178, however, median SI was slightly lower (112 vs. 123). The median T1-relaxation time was similar compared to all other tumor entities (1142 ms vs. 1159 ms). Lesion-to-Liver contrast of melanoma metastases was marginally higher compared to all other lesions (0.32 vs. 0.29).

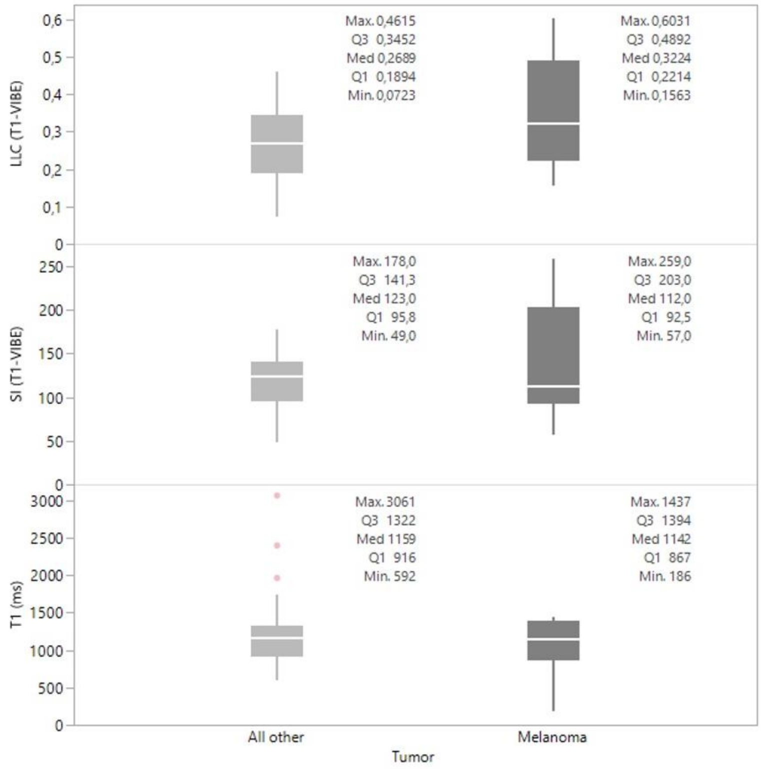

Supplement: Supplementary file 1 — Supplementary file1 (PDF 59 KB) [file 330_2023_9696_MOESM1_ESM.pdf]
